# Performance of commercially available anti-HDV enzyme-linked immunosorbent assays in Taiwan

**DOI:** 10.1186/s12985-020-01355-5

**Published:** 2020-06-16

**Authors:** Guan-Yu Lin, Yi-Le Wu, Cheng-Si Wang, Chia-Yun Ko, Chien-Hung Chen, Pei-Jer Chen, Po-Hsin Peng, Chao-Wei Hsu

**Affiliations:** 1 General Biologicals Corporation, Hsinchu, 30076 Taiwan; 2 Division of Hepato-Gastroenterology, Department of Internal Medicine, Kaohsiung Chang Gung Memorial Hospital and Chang Gung University College of Medicine, Kaohsiung, 83301 Taiwan; 3 Hepatitis Research Center, National Taiwan University, Taipei, 10002 Taiwan; 4 Department of Gastroenterology and Hepatology, Linkou Chang Gung Memorial Hospital and Chang Gung University College of Medicine, Taoyuan, 33305 Taiwan

**Keywords:** Kit evaluation, HDV, ELISA, Sensitivity, Specificity

## Abstract

**Background:**

Hepatitis D virus (HDV) infection is a major global health issue around the world. There are approximately 15–20 million individuals infected with HDV worldwide. HDV infection usually causes increased mortality compared with infection with hepatitis B virus (HBV) alone. However, testing for the detection of HDV is not widely available in Taiwan. Therefore, the General Biologicals Corporation (GB) HDV Ab kit was developed for detecting anti-HDV antibodies.

**Methods:**

A total of 913 serum and 462 EDTA-treated plasma samples were obtained from HBsAg-positive individuals in three hospitals in Taiwan from June 2014 to November 2017. We used three commercially available ELISA kits, DiaPro HDV Ab, DiaSorin ETI-AB-DELTAK-2 and GB HDV Ab, which were utilized strictly according to the instructions of the manufacturers.

**Results:**

A comparative study of the results from the GB HDV Ab kit and the other commercial ELISA kits (DiaPro and DiaSorin) was performed to determine their efficacy for anti-HDV detection. The results indicated that the sensitivity of the GB HDV Ab kit for serum and EDTA samples was 100% compared to that of the DiaPro and DiaSorin kits, whereas the specificity for serum and EDTA samples was 99.3 and 98.1%, respectively. In addition, the overall agreement of the results of the GB HDV Ab kit for the serum and EDTA samples was 99.3 and 98.3%, respectively. It is worth noting that the performance of the GB HDV Ab kit was not affected by interference from triglyceride, bilirubin, hemoglobin, or human anti-mouse antibody. The limit of detection of the GB HDV Ab kit is approximately 100-fold lower than that of the other two commercial kits.

**Conclusions:**

The GB HDV Ab kit, which presented equivalent sensitivity and specificity compared to both certified anti-HDV kits, would be a suitable kit for HDV diagnosis in Taiwan.

## Background

Hepatitis D virus (HDV) is a small single-stranded circular RNA with a negative polarity of approximately 1700 bases that produces hepatitis D antigen (HDAg) [1, 2]. Its replication is dependent on the hepatitis B virus (HBV) surface antigen (HBsAg) [3]. Coinfection with HDV and HBV usually leads to acute fulminant hepatitis, while superinfection can cause more severe hepatitis or cirrhosis than infection with HBV alone [4–6].

Previous epidemiological studies estimated that approximately 15–20 million people are infected with HDV worldwide [7]. However, these findings were challenged in 2018 by subsequent studies proposing that 48–74 million people were infected with HDV worldwide [8–10]. Although these estimates are still debated due to a lack of regional estimates [11, 12], recent studies have also shown that HDV infection is still endemic in most low-income countries [10]. Highly affected areas were the Western and Central Africa [13, 14], the Amazon basin [15] and the Mediterranean basin [16]. In particular, ∼60% of HBsAg-positive subjects were coinfected with HDV in Mongolia [17]. In Taiwan, the prevalence of HDV was approximately 4.4% among people infected with HBV [18].

However, the exact number of HDV infections is underestimated due to several factors. The first factor is the lack of knowledge of the regional HDV prevalence in low-income countries. Another is the rare systematic detection of anti-HDV antibodies in HBsAg-positive individuals, and the last is technical issues with enzyme immunoassays (EIAs), such as laboratory developed test (LDT) with lower sensitivity and specificity. These factors might lead to misdiagnosis and/or underestimation of HDV infection. Therefore, it is urgently necessary to establish sensitive and specific tools for HDV detection.

According to its genetic diversity, HDV has been divided into eight major genotypes (HDV-1 to HDV-8) [19, 20]. Among them, HDV-1, HDV-2, and HDV-4 are commonly found in Taiwan [18, 21]. The anti-HDV antibody is a useful marker to determine the activity of HDV infection [22]. Until 2018, enzyme immunoassays (EIAs) were the only technology used to detect anti-HDV antibodies on the market. Recently, many enzyme-linked immunosorbent assay (ELISA) kits have been developed to measure anti-HDV antibodies, such as DiaPro HDV Ab (DiaPro) and DiaSorin ETI-AB-DELTAK-2 (DiaSorin) [23, 24]. However, these diagnostic reagents are not licensed in Taiwan, so it is difficult to use them routinely in Taiwan. To date, domestically produced HDV diagnostic kits are mainly intended for research use only and usually have low sensitivity and specificity values. We successfully established a direct sandwich ELISA method (GB HDV Ab) to detect anti-HDV antibodies in human serum or plasma with ethylenediaminetetraacetic acid (EDTA).

In this study, 1375 HBsAg-positive specimens were examined to assess the performance of the GB HDV Ab kit and to compare the data from this kit with those obtained by using the two most commonly available ELISA kits from DiaPro and DiaSorin.

## Materials and methods

### Serum and EDTA-treated plasma specimen collection

A total of 913 serum and 462 EDTA-treated plasma samples were obtained from HBsAg-positive individuals in three hospitals in Taiwan from June 2014 to November 2017. The three hospitals were Kaohsiung Chang Gung Memorial Hospital (KCGMH), Linkou Chang Gung Memorial Hospital (LCGMH) and National Taiwan University Hospital (NTUH).

### Ethics

Ethical approval was obtained from the Institutional Review Board (IRB) of KCGMH (106-3378C), LCGMH (201700109B0) and NTUH (201612075DSB). Informed consent was sought and documented for all participants in this study.

### Performance of ELISA kits

Three commercially available ELISA kits, DiaPro HDV Ab (CE 0318), DiaSorin ETI-AB-DELTAK-2 (CE 0459) and GB HDV Ab (TFDA No. 005807), were utilized strictly according to the instructions of the manufacturers, as detailed in Table 1. The kits used in this study have different detection methods and use different HRP conjugates. According to the manufacturer’s guidelines, the cutoff value for the GB HDV Ab kit is defined as the mean value for the negative control + 0.11, and the cutoff for the DiaSorin kit is set as (0.5 × mean negative control) + (0.5 × mean positive control); the cutoff for the DiaPro kit is determined according to (mean negative control + mean positive control)/5. The positive and negative controls that were used were supplied by the manufacturer. Optical density (OD) was measured by an EMax Plus microplate reader (Molecular Devices), and the results were expressed as the cut-off index (COI = sample signal/cut-off value). HDV detection was considered positive for a COI value ≥1. The performance of the kits was evaluated in terms of sensitivity, specificity, and overall agreement with the 95% confidence intervals (95% CI) according to the CLSI EP12-A guidelines [25]. Sensitivity was calculated as true positives/(true positives + false negatives) × 100; specificity was calculated as true negatives/(true negatives + false positives) × 100; the overall agreement was calculated as (true positives + true negatives)/(true positives + false negatives + false positives + true negatives) × 100.

**Table 1:** Comparison of the three ELISA kits used for HDV detection

	GB HDV Ab	DiaPro HDV Ab	DiaSorin ETI-AB-DELTAK-2
**Manufacturer**	General Biological Corporation	DiaPro	DiaSorin
**Certification**	TFDA MHW Medical Device Manufacturing No. 005807	CE 0318	CE 0459
**Sample (volume)**	serum and EDTA-plasma(100 μl)	serum and EDTA-plasma (100 μl)	serum only (50 μl)
**Principle**	direct sandwich assay	two-step competitive assay	simultaneous competitive assay
**Adsorbed Antigen**	recombinant HDV antigen	recombinant HDV antigen	recombinant HDV antigen
**HRP Conjugate**	HDV small antigen	polyclonal Ab against HDV	human anti-HD Fab
**Incubation Time**	105 min (60, 30, 15 min)	140 min (60, 60, 20 min)	210 min (180, 30 min)
**Incubation Temperature**	37 °C	37 °C or room temperature	37 °C orroom temperature
**Reader Wavelength**	450 nm (reading) 650 nm (blanking, if possible)	450 nm (reading) 620–630 nm (blanking, if possible)	450 nm (reading) 630 nm (blanking)
**Control Well(s)**	6 (Blank*1, NC*3, PC*2)	7 (Blank*1, NC*3, CAL*2, PC*1)	6 (Blank*1, NC*3, PC*2)

### Interference testing

Interference testing was performed according to the CLSI EP07-A2 guidelines [26]. Briefly, 200 mg/dl triglyceride (TG) from an in-house serum sample with a high triglyceride titer (~ 400 mg/dl) was used as the stock; 0.4 mg/dl bilirubin (BL), which was obtained from Sigma, 5.5 g/dl hemoglobin (HB), which was obtained from healthy donors, 17 ng/ml human anti-mouse antibody plasma (HAMA), which was obtained from Meridian Life Science, and a three-fold dilution of a multi-analyte positive control (MAPC), which was obtained from SeraCare (Accurun Series 2700) and contained anti-HIV-1/2, anti-HTLV-I/II, anti-HBc, anti-HCV, anti-CMV, anti-*Treponema pallidum*, and HBsAg, was spiked into the serum and EDTA-plasma from HDV-positive patients.

### The detection limit of the ELISA kits

The detection limits of the three commercially available ELISA kits were determined by using decreasing concentrations of anti-HDV in normal human plasma (NHP) or guinea pig sera. Anti-HDV was obtained from two standard sensitivity panels, including ACCURUNⓇ 127 Anti-Hepatitis Delta Positive Control (CE-IVD) from SeraCare Life Sciences and polyclonal anti-HDV from guinea pig.

### Statistical analysis

All calculations were performed using Microsoft Excel 2016. Statistical significance (*P* values < 0.05) was assessed by the two-tailed Student’s t-test. The sensitivity, specificity, and overall agreement with the 95% CI were estimated for each kit.

## Results

**Fig. 1:**
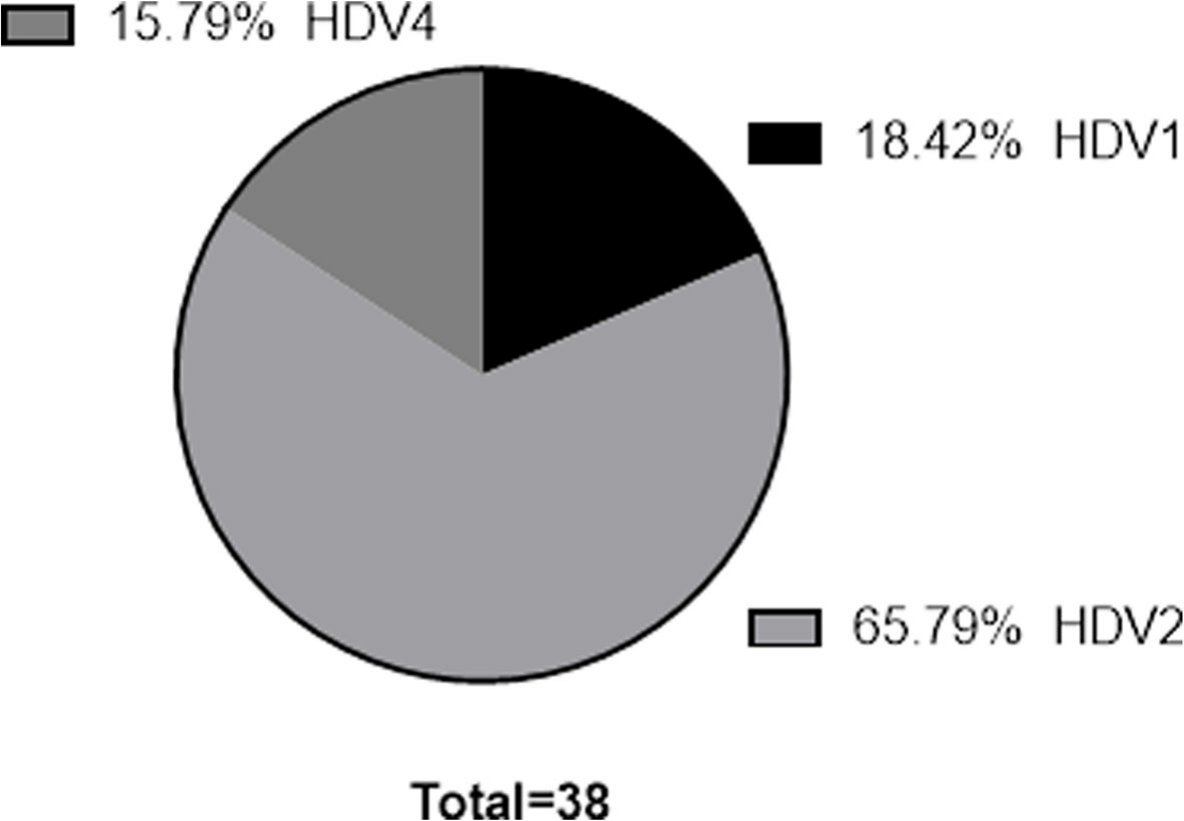
Comparison of the detection limit of the GB, DiaPro and DiaSorin kits. Anti-HDV antibodies from human plasma (**a**) and guinea pig sera (**b**) were serially 2-fold diluted with normal human plasma and detected by the three commercial kits

In the current study, we developed a direct sandwich GB HDV Ab kit, which can detect total anti-HDV antibodies. We determined the detection limits of the GB HDV Ab kit and commercial ELISA kits. Anti-HDV antibodies from humans and guinea pigs were serially 2-fold diluted with normal human plasma (NHP). The results showed that the GB kit had superior analytical sensitivity compared to the DiaPro and DiaSorin kits. The detection limit of the GB HDV Ab kit for ACCURUN 127 was 2^11^-fold, which was better than that of the DiaPro (2^5^-fold) and DiaSorin (2^9^-fold) kits; for polyclonal anti-HDV antibodies from guinea pig, the detection limit of the GB HDV Ab kit was 2^9^-fold, which was better than that of the DiaPro (2^7^-fold) and comparable to that of the DiaSorin (2^9^-fold) kits (Fig. 1).

In the current study, a total of 913 serum specimens and 462 EDTA-treated plasma samples from HBV-infected individuals from three hospitals in Taiwan obtained from June 2014 to November 2017 were tested with commercially available HDV detection ELISA kits from GB, DiaPro and DiaSorin, and the results are summarized in Table 2. For serum samples, it was evident that the GB HDV Ab kit had a similar performance, for which the specificity was 97.3% and the sensitivity was 100% compared to the DiaPro kit. The overall agreement of the GB HDV Ab kit results for the serum samples was 97.6%. Moreover, the GB HDV Ab kit also had good performance for the EDTA-treated plasma samples, for which the specificity was 97.2% and the sensitivity was 100%. The overall agreement of the results for the GB HDV Ab kit was 97.4%. The data indicated that the GB kit had a very similar performance compared to that of the DiaPro kit. However, 22 serum samples and 12 EDTA-treated plasma samples showed inconsistent results between the GB and DiaPro kits. Therefore, we used a third commercial kit, the DiaSorin ELISA kit, to confirm the positive or negative results for these inconsistent samples. The results showed that 15 serum samples and 4 EDTA-treated plasma samples were HDV-positive samples, and the results for one sample for the DiaSorin kit were equivocal. The equivocal result was excluded from the calculations. By doing so, the specificity of the GB HDV Ab kit for the serum and EDTA samples was determined to be 99.3 and 98.1%, respectively (Table 3). The sensitivity of the GB HDV Ab kit for the serum and EDTA samples was 100%. The overall agreement of the results for the GB HDV Ab kit for the serum and EDTA samples was 99.3 and 98.3%, respectively. These results were comparable to those obtained with the commercial ELISA kits employed in this study.

**Table 2:** Performance of the GB kit compared to the DiaPro kit

		DiaPro					
		Serum		Total no.	EDTA-treated plasma		Total no.
		Positive	Negative		Positive	Negative	
GB	Positive	86	22	108	26	12	38
	Negative	0	805	805	0	424	424
	Total no.	86	827	913	26	436	462
Sensitivity (%)		100.0 (95.7 ~ 100.0)			100.0 (87.1 ~ 100.0)		
Specificity (%)		97.3 (96.0 ~ 98.2)			97.2 (95.3 ~ 98.4)		
Overall Agreement (%)		97.6 (95.5 ~ 99.3)			97.4 (94.9 ~ 99.1)

**Table 3:** Performance of the GB kit compared to the DiaPro + DiaSorin kits

		DiaPro + DiaSorin					
		Serum		Total no.	EDTA-treated plasma		Total no.
		Positive	Negative		Positive	Negative	
GB	Positive	101	6	107	30	8	38
	Negative	0	805	805	0	424	424
	Total no.	101	811	912	30	432	462
Sensitivity (%)		100.0 (96.3 ~ 100.0)			100.0 (88.7 ~ 100.0)		
Specificity (%)		99.3 (98.4 ~ 99.7)			98.1 (96.4 ~ 99.1)		
Overall Agreement (%)		99.3 (97.1 ~ 100.0)			98.3 (95.6 ~ 100.0)		

**Fig. 2:**
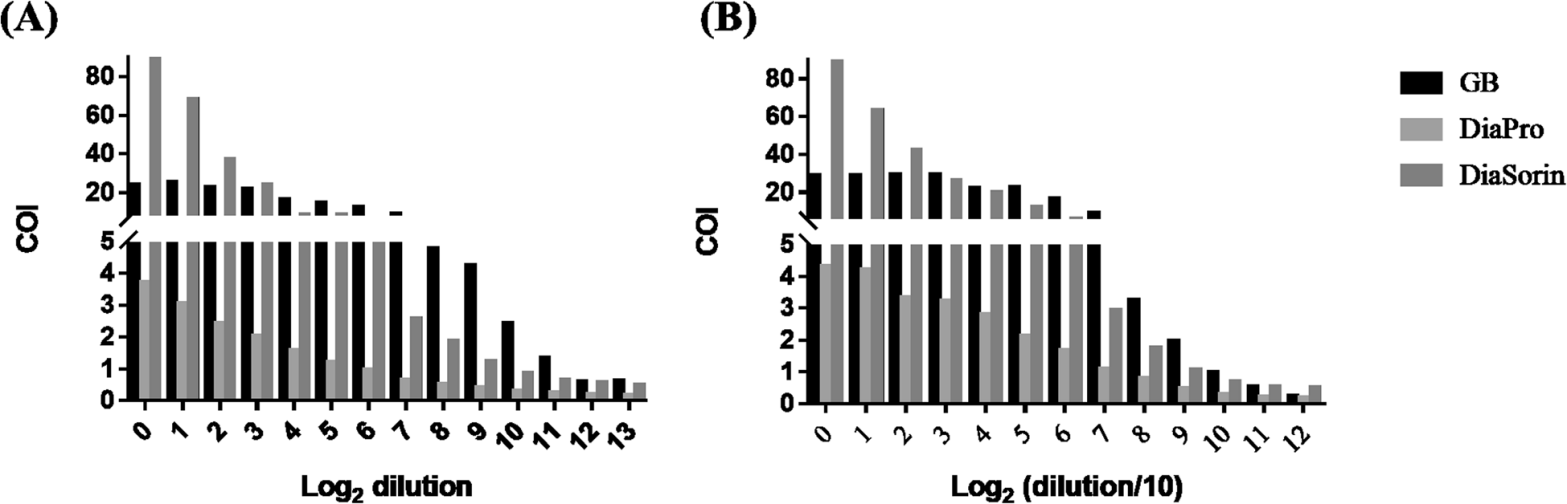
Distribution of HDV subtypes among the 38 HDV-positive samples The HDV samples were analyzed by sequencing. The sequences were aligned to confirm the HDV subtype

In addition, we also analyzed the genotypes of 38 HDV-positive samples from patients with HBV infection using PCR and DNA sequencing. A prevalence of 18.4% for the HDV-1 genotype (*n* = 7), 65.8% for the HDV-2 genotype (*n* = 25) and 15.8% for the HDV-4 genotype (*n* = 6) was observed. All the samples were positive for HDV antibodies, as detected by the GB HDV Ab kit (Fig. 2). These data indicate that the GB HDV Ab kit was able to detect antibodies from specimens containing HDV-1, HDV-2 and HDV-4, which are commonly found in Taiwan.

To determine which analytes might interfere with the performance of the GB HDV Ab kit, interference assays were performed. Interference test samples were generated by spiking potential interfering materials into serum and EDTA-treated plasma containing anti-HDV plasma (ACCURUN 127) (Table 4). The results showed that none of the interfering materials in serum and none of the materials except TG, BL and HAMA in EDTA-treated plasma affected the performance of the GB HDV Ab kit (*p* < 0.05). However, HB and MAPC in EDTA-treated plasma might affect the values of the COI determined by the GB HDV Ab kit.

**Table 4:** Performance of the GB kit in the presence of interfering compounds

Specimen	Interfering compound	mean COI	±	std. Dev.	***p***-value
**Serum**	TG (−)	3.65	±	0.60	0.87
	TG (+)	3.59	±	0.09	
	BL (−)	2.95	±	0.83	0.77
	BL (+)	2.75	±	0.67	
	HB (−)	3.85	±	0.38	0.62
	HB (+)	3.68	±	0.40	
	HAMA (−)	3.60	±	0.16	0.43
	HAMA (+)	3.80	±	0.35	
	MAPC (−)	3.26	±	0.49	0.20
	MAPC (+)	3.89	±	0.52	
**EDTA-treated plasma**	TG (−)	4.09	±	0.60	0.24
	TG (+)	3.57	±	0.23	
	BL (−)	3.65	±	0.84	0.48
	BL (+)	3.24	±	0.39	
	HB (−)	4.59	±	0.26	0.03
	HB (+)	3.90	±	0.26	
	HAMA (−)	4.67	±	0.27	0.23
	HAMA (+)	4.43	±	0.11	
	MAPC (−)	4.09	±	0.09	0.01
	MAPC (+)	5.02	±	0.30	

*TG* triglyceride, *BL* bilirubin, *HB* hemoglobin, *HAMA* human anti-mouse antibody plasma, *MAPC* multi-analyte positive control (SeraCare Accurun Series 2700)

**Fig. 3:**
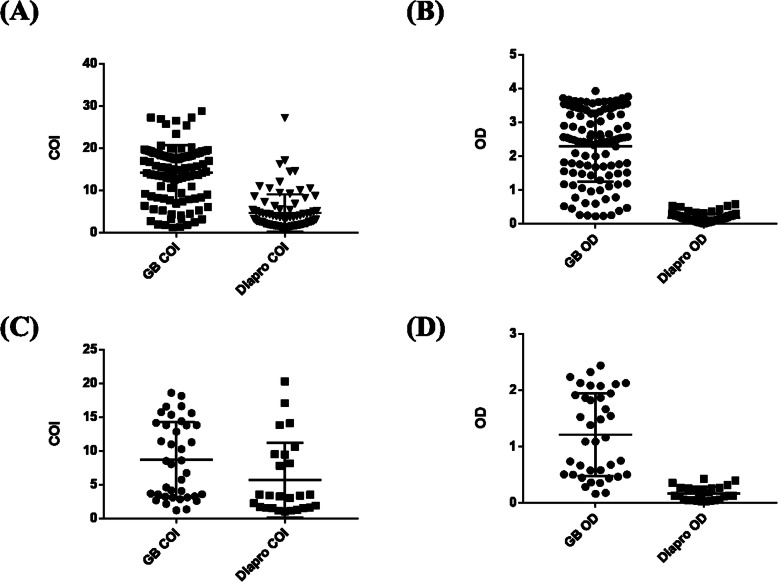
Distribution of the results for the clinical specimens tested with the GB and DiaPro kits. Results for the HDV-positive serum samples expressed as the COI (**a**) or OD (**b**). Results for the HDV-positive EDTA-treated plasma expressed as the COI (**c**) or OD (**d**)

In addition, the detection ranges for the COI and OD values of the positive samples with serum and EDTA-treated plasma obtained with the GB HDV Ab kit were wider than those of the DiaPro kit (Fig. 3). These results indicated that the GB kit could detect lower levels of anti-HDV antibodies and also had a wider detection range for HDV antibodies without dilution.

## Discussion

In this study, the performance of three commercial HDV ELISA kits (GB, DiaPro, and DiaSorin) was investigated for serum and EDTA-treated plasma. An important issue that was encountered was the discordance between the results of the GB and DiaPro kits. Therefore, another available kit, DiaSorin, was used to resolve this issue. Among 913 serum samples, 86 HDV-positive samples and 827 HDV-negative samples were determined by the DiaPro kit. On the other hand, 108 HDV-positive samples and 805 HDV-negative samples were determined by the GB HDV Ab kit. Therefore, the 22 inconsistent samples were reconfirmed with the DiaSorin kit, resulting in 15 HDV-positive samples, 6 HDV-negative samples, and one equivocal result. Then, we assumed that the real status of the 912 serum samples reflected 101 HDV-positive samples and 811 HDV-negative samples. By doing so, the overall sensitivity and specificity of the GB HDV Ab kit were shown to be 100 and 99.3%, whereas those of the DiaPro kit were shown to be 85.1 and 100%, respectively. The results for the serum samples indicated that the performance of the GB HDV Ab kit was better than that of the DiaPro kit. Among the 462 EDTA samples, 26 HDV-positive samples and 436 HDV-negative samples were determined by the DiaPro kit. However, 38 HDV-positive samples and 424 HDV-negative samples were determined by the GB HDV Ab kit. Therefore, the 12 inconsistent samples were also reconfirmed by using the DiaSorin kit, which resulted in 4 HDV-positive samples and 8 HDV-negative samples. Then, we assumed that the 462 EDTA samples included 30 HDV-positive samples and 432 HDV-negative samples. Therefore, the overall sensitivity and specificity of the GB HDV Ab kit were 100 and 98.1%, respectively, whereas those of the DiaPro were 86.7 and 100%, respectively. Therefore, the present study revealed the better performance of the GB HDV Ab kit in terms of specificity and sensitivity compared to that of the DiaPro kit. In addition, the detection limit of the GB HDV Ab kit was better than that of the DiaPro and DiaSorin (Fig. 1). Therefore, the 6 negative serum and 8 negative plasma samples by DiaSorin might contain lower concentration of anti-HDV antibodies and only could be determined by GB HDV Ab kit due to the lower detection limit of GB kit. Further study is needed to address these questions.

In addition, the ELISA assay is usually affected by binding to natural unknown proteins, such as human anti-animal antibodies [27]. Therefore, we also used interference assays to determine the performance of the GB HDV Ab kit in the presence of interfering materials. The kit performance did not significantly change in the presence of five different interfering materials in serum except for HB and MAPC, for which the results for EDTA-treated plasma showed statistically significant variation (*p* < 0.05). Although not yet understood, the slight background observed in EDTA-plasma with hemoglobin (HB) or MAPC might be due to unspecific binding of the antibodies. The results indicated that the GB HDV Ab kit is suitable for detecting anti-HDV antibodies in serum samples.

## Conclusion

The GB HDV Ab kit can successfully detect anti-HDV antibodies from patients infected with HDV genotypes 1, 2 and 4, which are commonly found in Taiwan. The GB kit has higher sensitivity and specificity than commercial ELISA kits. In addition, the GB HDV Ab kit showed improved performance during interference testing of serum samples, a wider detection range and a lower detection limit than commercial kits. The results indicated that the GB HDV Ab kit might be suitable for screening patients infected with HDV.

## Data Availability

The datasets used and/or analysed during the current study are available from the corresponding author on reasonable request.

## Abbreviations

BL: Bilirubin
CI: Confidence intervals
COI: Cut-off index
EDTA: Ethylenediaminetetraacetic acid
ELISA: Enzyme-linked immunosorbent assay
HAMA: Human anti-mouse antibody plasma
HB: Hemoglobin
HBsAg: Hepatitis B virus surface antigen
HDAg: Hepatitis D virus antigen
HBV: Hepatitis B virus
HDV: Hepatitis D virus
IRB: Institutional Review Board
KCGMH: Kaohsiung Chang Gung Memorial Hospital
LCGMH: Linkou Chang Gung Memorial Hospital
MAPC: Multi-analyte positive control
NHP: Normal human plasma
NTUH: National Taiwan University Hospital
OD: Optical Density
TG: Triglyceride

## References

[1] Taylor JM (2006). Hepatitis delta virus. Virology..

[2] Taylor JM, Hepatitis D. Virus Replication. Cold Spring Harb Perspect Med. 2015;5(11):a021568. 10.1101/cshperspect.a021568 PMC4632862 26525452

[3] Rizzetto M, Canese MG, Arico S, Crivelli O, Trepo C, Bonino F, Verme G (1977). Immunofluorescence detection of new antigen-antibody system (delta/anti-delta) associated to hepatitis B virus in liver and in serum of HBsAg carriers. Gut..

[4] Thomas E, Yoneda M, Schiff ER (2015). Viral hepatitis: past and future of HBV and HDV. Cold Spring Harb Perspect Med..

[5] Sureau C, Negro F (2016). The hepatitis delta virus: replication and pathogenesis. J Hepatol.

[6] Negro F (2014). Hepatitis D virus coinfection and superinfection. Cold Spring Harb Perspect Med.

[7] Lempp FA, Ni Y, Urban S (2016). Hepatitis delta virus: insights into a peculiar pathogen and novel treatment options. Nat Rev Gastroenterol Hepatol.

[8] Chen HY, Shen DT, Ji DZ, Han PC, Zhang WM, Ma JF, Chen WS, Goyal H, Pan S, Xu HG. Prevalence and burden of hepatitis D virus infection in the global population: a systematic review and meta-analysis. Gut. 2018. 10.1136/gutjnl-2018-316601. 10.1136/gutjnl-2018-316601 30228220

[9] Shen DT, Ji DZ, Chen HY, Goyal H, Pan S, Xu HG. Hepatitis D: not a rare disease anymore: global update for 2017-2018. Gut. 2019. 10.1136/gutjnl-2019-318691. 10.1136/gutjnl-2019-318691 30967414

[10] Miao Z, Zhang S, Ou X, Li S, Ma Z, Wang W, Peppelenbosch MP, Liu J, Pan Q. Estimating the global prevalence, disease progression and clinical outcome of hepatitis delta virus infection. J Infect Dis. 2019. 10.1093/infdis/jiz633. 10.1093/infdis/jiz633 PMC7184909 31778167

[11] Stockdale AJ, Kreuels B, Henrion MRY, Giorgi E, Kyomuhangi I, Geretti AM. Hepatitis D prevalence: problems with extrapolation to global population estimates. Gut. 2018. 10.1136/gutjnl-2018-317874. 10.1136/gutjnl-2018-317874 30567743

[12] Wedemeyer H, Negro F. Devil hepatitis D: an orphan disease or largely underdiagnosed? Gut. 2018. 10.1136/gutjnl-2018-317403. 10.1136/gutjnl-2018-317403 30368454

[13] Groc S, Abbate JL, Le Gal F, Gerber A, Tuaillon E, Albert JL, Nkoghe D, Leroy EM, Roche B, Becquart P (2019). High prevalence and diversity of hepatitis B and hepatitis delta virus in Gabon. J Viral Hepat.

[14] Makiala-Mandanda S, Le Gal F, Ngwaka-Matsung N, Ahuka-Mundeke S, Onanga R, Bivigou-Mboumba B, Pukuta-Simbu E, Gerber A, Abbate JL, Mwamba D, Berthet N, Leroy EM, Muyembe-Tamfum JJ, Becquart P (2017). High prevalence and diversity of Hepatitis viruses in suspected cases of yellow fever in the Democratic Republic of Congo. J Clin Microbiol.

[15] Scarponi CFO, Silva R, Souza Filho JA, Guerra MRL, Pedrosa MAF, Mol MPG (2019). Hepatitis Delta prevalence in South America: a systematic review and Meta-analysis. Rev Soc Bras Med Trop.

[16] Amini N, Alavian SM, Kabir A, Aalaei-Andabili SH, Saiedi Hosseini SY, Rizzetto M (2013). Prevalence of hepatitis d in the eastern mediterranean region: systematic review and meta analysis. Hepat Mon.

[17] Chen X, Oidovsambuu O, Liu P, Grosely R, Elazar M, Winn VD, Fram B, Boa Z, Dai H, Dashtseren B, Yagaanbuyant D, Genden Z, Dashdorj N, Bungert A, Dashdorj N, Glenn JS (2017). A novel quantitative microarray antibody capture assay identifies an extremely high hepatitis delta virus prevalence among hepatitis B virus-infected mongolians. Hepatology..

[18] Lin HH, Lee SS, Yu ML, Chang TT, Su CW, Hu BS, Chen YS, Huang CK, Lai CH, Lin JN, Wu JC (2015). Changing hepatitis D virus epidemiology in a hepatitis B virus endemic area with a national vaccination program. Hepatology..

[19] Le Gal F, Gault E, Ripault MP, Serpaggi J, Trinchet JC, Gordien E, Deny P (2006). Eighth major clade for hepatitis delta virus. Emerg Infect Dis.

[20] Deny P (2006). Hepatitis delta virus genetic variability: from genotypes I, II, III to eight major clades?. Curr Top Microbiol Immunol.

[21] Kao JH, Chen PJ, Lai MY, Chen DS (2002). Hepatitis D virus genotypes in intravenous drug users in Taiwan: decreasing prevalence and lack of correlation with hepatitis B virus genotypes. J Clin Microbiol.

[22] Sahin A, Gurocak S, Tunc N, Demirel U, Poyrazoglu OK, Akbulut H, Yalniz M, Toraman ZA, Bahcecioglu IH (2018). Anti-HDV seroprevalance among patients with previous HBV infection. North Clin Istanb.

[23] Tajbakhsh E, Tajbakhsh S, Yaghobi R, Momeni M, Tajbakhsh F, Hamedi S (2011). Serological survey of HDV-Ab in HBsAg positive blood donors in Shahrekord. Iran Afr J Microbiol Res.

[24] Rocco C, Bonavolta R, Vallefuoco L, Braschi U, Sorrentino R, Terracciano D, Portella G. Comparison of anti-hepatitis D virus (HDV) ETI-AB-DELTAK-2 assay and the novel LIAISON(R) XL MUREX anti-HDV assay in the diagnosis of HDV infection. Diagn Microbiol Infect Dis. 2019;95(4):114873. 10.1016/j.diagmicrobio.2019.114873 31473034

[25] CLSI (2008). User Protocol for Evaluation of Qualitative Test Performance; Approved Guideline-Second Edition. CLSI Document EP12-A2.

[26] CLSI (2005). Interference Testing in Clinical Chemistry; Approved Guideline. CLSI Document EP07.

[27] Tate J, Ward G (2004). Interferences in immunoassay. Clin Biochem Rev.

